# Supplementary material for: Real-time observation of epitaxial graphene domain reorientation
Source: Nat Commun. 2015 Apr 20;6:6880. doi: 10.1038/ncomms7880 (PMC4411305; doi:10.1038/ncomms7880)
Supplement: Supplementary Figure, Supplementary Table, Supplementary Notes and Supplementary References — Supplementary Figure 1, Supplementary Table 1, Supplementary Notes 1-3 and Supplementary References [file ncomms7880-s1.pdf]

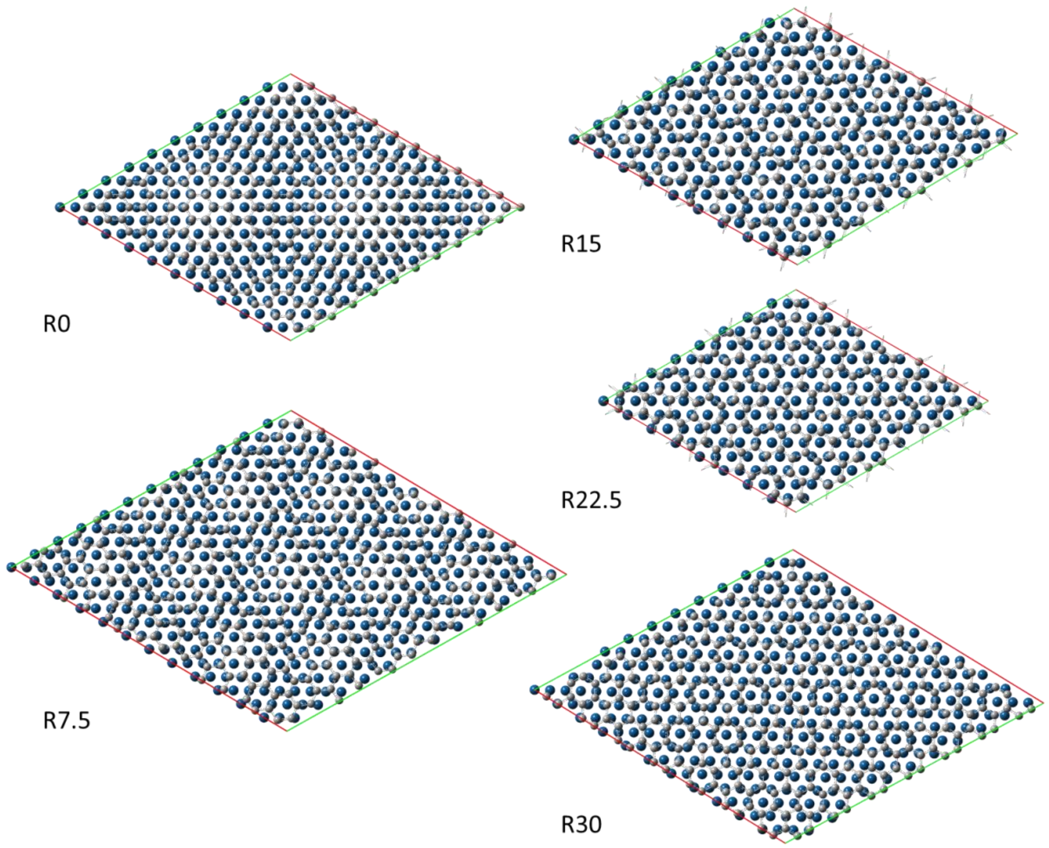

Supplementary Figure 1: The optB86b-vdW optimized R0, R7.5, R15, R22.5, and R30 structures.

Supplementary Table 1: DFT Results. The optB86b-vdW predicted graphene deformation energies (bending energies), absolute and relative binding energies and difference in the predicted values using a 1x1x1 and a 2x2x1 k-point grid (ΔE/Δk).

	Graphene Deformation Energy per C Atom (meV)			Binding Energy per C Atom (meV)			Relative Binding Energy per C Atom (meV)	
k-points	1x1x1	2x2x1	ΔE/Δk	1x1x1	2x2x1	ΔE/Δk	1x1x1	2x2x1
R0	0.70	0.63	-0.07	-77.11	-76.68	0.43	0.00	0.00
R7.5	0.87	0.88	0.00	-77.82	-77.39	0.43	-0.71	-0.71
R15	0.23	0.24	0.01	-76.36	-76.48	-0.12	0.75	0.19
R22.5	0.10	0.12	0.01	-77.04	-76.62	0.42	0.07	0.05
R30	0.33	0.33	0.00	-76.93	-76.19	0.74	0.18	0.48

Supplementary Note 1

One might be concerned that edge energies would be important in determining the chemical potential differences, as they are in standard Ostwald ripening. However, we observed no influence of R0 island size on ripening velocity, which suggests that edge energies are not a significant factor. The R0 areas varied in size from 17 μm^2 to 90 μm^2 among the various experiments. For example, in one experiment, a R14 domain was stationary at 1010 °C, while the R0 velocity was 0.20 nm/s and had an area of 76 μm^2. In another experiment, a R14 domain was stationary at 1000 °C, while the R0 velocity was 0.15 nm/s and had an area of 22 μm^2. Their velocity ratio was ~1.3; if the velocity was influenced by edge energies, the velocity ratio should depend on the ratio of the island radii, ~1.9. Instead, the velocities can be accounted for by the ratio of the kinetic coefficients, *B*(1010 °C)/*B*(1000 °C) ≈ 1.2. This indicates that the R0 velocity was independent of island size for these island sizes.

Supplementary Note 2

We further support the reported differences in chemical potential by analyzing the curvature of the moving domain boundary shown in Fig. 1g in the main manuscript. Initially, the boundary has a structure in which the curvature changes sign, i.e., the curvature inverts, an artifact attributed to the initial growth of the island. When the boundary began to move, the curvature changed such that the boundary extended into the R12 domain only (Fig. 1g, middle frame). This curvature is attributed to pinning by the lower R0 domain. Indeed, as the bottom end of the boundary moved beyond the R0 domain, the boundary straightened (Fig. 1g, right frame). The radius of curvature of the boundary while it was moving, but partially pinned, was ~9 μm, as seen in the middle frame of Fig. 1g. We use this curvature to estimate the difference in chemical potential between the two domains.

Consider an idealized situation in which two phases with different chemical potentials meet at a boundary with both ends pinned. The boundary will move in response to the chemical potential difference, but because its ends are pinned, it will become curved and resultantly lengthen. Equilibrium is achieved when the free energy cost of further increasing the boundary length is equal to the free energy gain by transferring mass from the phase of higher to lower chemical potential, in which the difference in chemical potential is given by

$$\Delta\mu = \frac{\Omega\gamma}{R} \tag{1}$$

where Ω is the atomic area, γ is the domain boundary energy per unit length, and R is the radius of curvature. Although the boundary is moving in the example shown in Fig. 1g, we use the equilibrium picture to estimate a lower bound of the difference in chemical potentials, i.e., if the boundary was pinned completely, the radius of curvature would decrease, and thus increase $\Delta\mu$. *Ab-initio* calculations previously estimated γ as a function of misorientation between two graphene domains. For a misorientation of 1°, we extrapolate the results from Supplementary Reference 5, which gives $\gamma \approx 0.05$ eV/Å. Given the graphene atomic density of $3.8*10^{15}$ cm^{-2} and the 9 μm radius of curvature between the R11 and R12 domain, the resulting difference in chemical potential is ~0.002 meV/C (Supplementary equation (1)). As a comparison, the velocity analysis reported in Fig. 3d gives $\Delta\mu$ = 0.007 meV between R11 and R12. As expected, this

result is higher than that obtained from the domain boundary motion. More importantly, the domain boundary analysis supports the order of magnitude of the reported values in Fig. 3d.

Supplementary Note 3

Consider the curvature around atom *i* and assume atom *i* and its three nearest-neighbors (NN) lie on a paraboloid (~sphere with radius $r = 1/k$ where *k* is the curvature) with a coordinate system centered at atom *i* and the x-y plane tangential to the paraboloid. Consider everything to be rather flat and approximate lengths by their projections, $z = (1/2)(k_1 x^2 + k_2 y^2)$ and assume $k_1 = k_2 = k = 1/r$. Let the *x*-axis point to a neighboring atom's projection. This neighbor (and all other nearest neighbors) are higher than atom *i* by

$$\Delta h_i = z_i = \frac{1}{2} k_i d^2 \quad (2)$$

with C-C distance *d* = 1.42 Å. The curvature at atom *i* is

$$k_i = 2z_i/d^2 \text{ or } k_i^2 = 4z_i^2/d^4. \quad (3)$$

From continuum theory, the energy due to bending is[6]

$$E_b = \frac{1}{2} \lambda \int K^2 dS \quad (4)$$

where λ is the mean bending rigidity, ~1.4 eV[7], *K* is the mean curvature, and noting that the Gaussian bending vanishes for a 2D, periodic sheet[7]. Converting the integral to a sum over *i* gives

$$E_b = \frac{1}{2} \lambda \sum k_i^2 S_0 \quad (5)$$

where the footprint of a C atom $S_0 = 3d^2\sqrt{3}/4$. Substituting for k_i (Supplementary equation (3)) and S_0,

$$E_b = \frac{3\sqrt{3}\lambda}{2} \sum (z_i/d)^2, \quad (6)$$

or the bending energy per atom

$$\frac{E_b}{N} = \frac{3\sqrt{3}\lambda}{2N} \sum (z_i/d)^2. \quad (7)$$

Differentiating with respect to $z_i$gives the expression for the force on each atom specified in the text

Supplementary References

[1] Arnoult, W. J. & McLellan, R. B. The solubility of carbon in rhodium, ruthenium, iridium, and rhenium. *Scripta Metall*. **6,** 1013-1018 (1972).
[2] Criscione, J. M., Volk, J. F. & Smith, A. W. Protection of graphite from oxidation at 2100 C. *AIAA J.* **4,** 1791-1797 (1966).
[3] McCarty, K. F., Feibelman, P. J., Loginova, E. & Bartelt, N. C. Kinetics and thermodynamics of carbon segregation and graphene growth on Ru(0001). *Carbon* **47,** 1806-1813 (2009).
[4] Starodub, E., Bartelt, N. C. & McCarty, K. F. Oxidation of graphene on metals. *J. Phys. Chem.* **114,** 5134-5140 (2010).
[5] Yazyev, O. V. & Louie, S. G. Topological defects in graphene: Dislocations and grain boundaries. *Phys. Rev. B* **81,** 195420 (2010).
[6] Landau, L. D. & Lifshitz, E. M. *Theory of Elasticity* (Pergamon Press, Oxford, UK, 1986).
[7] Chen, S. & Chrzan, D. C. Continuum theory of dislocations and buckling in graphene. *Phys. Rev. B* **84,** 214103 (2011).
